# A Qualitative Research to Examine Experiences of Turkish Women with Breast Cancer

**DOI:** 10.1192/j.eurpsy.2022.1699

**Published:** 2022-09-01

**Authors:** C. Yastibaş, G. Dirik, İ.G. Yilmaz-Karaman

**Affiliations:** 1 Dokuz Eylül University, Psychology Department, İzmir, Turkey; 2 Eskişehir Osmangazi University, Faculty of Medicine, Psychiatry Department, Eskişehir, Turkey

**Keywords:** breast cancer, Qualitative research, post-traumatic growth, Distress

## Abstract

**Introduction:**

Breast cancer is a serious public health problem and one out of every 4 women diagnosed with cancer is breast cancer. Although the survival rate has increased due to advances in diagnosis and treatment, getting a cancer diagnosis is a highly stressful life event and seriously affects the lives of patients.

**Objectives:**

Therefore, the aim of this qualitative study is to explore the experiences of women with breast cancer.

**Methods:**

Data were gathered using semi-structured forms, in-depth interviews with 7 patients aged between 29 and 64 who had been diagnosed with breast cancer in 2017 and after. All interviews were tape-recorded and the themes have resulted in analyzing the content of the recorded data.

**Results:**

It has been determined that women have difficulties in getting information from healthcare professionals, emotional supports from their partners and family members, dealing with losses in roles and femininity, and coping with intrusive thoughts. However, it has been highlighted that women have experienced some positive changes in certain areas such as deeper interpersonal relationships with others, appreciating health and life.

**Conclusions:**

As a result of the study, it has been thought that it is important to reduce the distress level of women with breast cancer related to their needs and difficulties and to encourage their positive changes. Besides, working in cooperation with healthcare professionals responsible for their treatment will be beneficial to reduce the distress level of the women.

**Disclosure:**

No significant relationships.

